# Microbial-Assisted Wheat Iron Biofortification Using Endophytic *Bacillus altitudinis* WR10

**DOI:** 10.3389/fnut.2021.704030

**Published:** 2021-08-03

**Authors:** Zhongke Sun, Zonghao Yue, Hongzhan Liu, Keshi Ma, Chengwei Li

**Affiliations:** ^1^College of Life Science and Agronomy, Zhoukou Normal University, Zhoukou, China; ^2^College of Biological Engineering, Henan University of Technology, Zhengzhou, China

**Keywords:** iron biofortification, wheat grain, endophyte, *Bacillus* spp., field study

## Abstract

Microbial-assisted biofortification attracted much attention recently due to its sustainable and eco-friendly nature for improving nutrient content in wheat. An endophytic strain *Bacillus altitudinis* WR10, which showed sophistical regulation of iron (Fe) homeostasis in wheat seedlings, inspired us to test its potential for enhancing Fe biofortification in wheat grain. In this study, assays *in vitro* indicated that WR10 has versatile plant growth-promoting (PGP) traits and bioinformatic analysis predicted its non-pathogenicity. Two inoculation methods, namely, seed soaking and soil spraying, with 10^7^ cfu/ml WR10 cells were applied once before sowing of wheat (*Triticum aestivum* L. cv. Zhoumai 36) in the field. After wheat maturation, evaluation of yield and nutrients showed a significant increase in the mean number of kernels per spike (KPS) and the content of total nitrogen (N), potassium (K), and Fe in grains. At the grain filling stage, the abundance of *Bacillus* spp. and the content of N, K, and Fe in the root, the stem, and the leaf were also increased in nearly all tissues, except Fe in the stem and the leaf. Further correlation analysis revealed a positive relationship between the total abundance of *Bacillus* spp. and the content of N, K, and Fe in grains. Seed staining confirmed the enhanced accumulation of Fe, especially in the embryo and the endosperm. Finally, using a hydroponic coculture model, qPCR quantification indicated effective colonization, internalization, translocation, and replication of strain WR10 in wheat within 48 h. Collectively, strain WR10 assisted successful Fe biofortification in wheat in the field, laying a foundation for further large-scale investigation of its applicability and effectiveness.

## Introduction

Iron (Fe) is an essential trace element for the health of both plants and humans; however, most Fe in the soil is not readily accessible to plants (ferric form, Fe^3+^), resulting in low bioavailability (1). Furthermore, Fe deficiency is one of the most prevalent forms of malnutrition in the world, and one-fifth of the population in China suffers from Fe deficiency (http://www.chinacdc.cn). The long-term acquisition of Fe by humans is mainly through food, highlighting the importance of Fe content in staple crops (2). As one of the most important food crops in the world, wheat provides various nutrients, including Fe, to hundreds of millions of people. The HarvestPlus project suggested that wheat grains should contain 59 mg/kg of Fe to meet the dietary Fe needs of adults (3); however, the average Fe content of 198 wheat varieties was only 29.1 mg/kg in France (4), and that of 260 varieties in the Huanghuai wheat region of China was only 22.2 mg/kg (5). Therefore, Fe deficiency remains one of the most serious global nutritional problems.

Several approaches have been developed to overcome Fe deficiency in humans (6); however, only biofortification, a process of breeding nutrients into food crops, is considered to be a sustainable strategy for tackling malnutrition, especially for those who have limited access to diverse diets or fortified foods. Indeed, the biofortification of staple crops is an evidence-based and cost-effective method to address malnutrition in tens of millions of people (https://www.harvestplus.org/biofortification-nutrition-revolution-now). In general, Fe biofortification can be achieved mainly by plant breeding, transgenic techniques, or agronomic practices (7). Therefore, wheat Fe biofortification is an urgent and economically important task (8). Promoting root absorption of Fe from the soil and increasing Fe accumulation in grains have become the most fundamental, efficient, and sustainable methods of wheat biofortification (9, 10); however, so far, progress in wheat Fe biofortification by traditional plant breeding has not been as successful as in other crops (11). Although transgenic techniques have developed high-iron genotypes, their release is still restricted. Agronomic practices, mainly foliar application of Fe-containing chemical fertilizers, are currently the major methods used for wheat; however, these practices are unappealing due to mineral unsustainability and potential adverse effects on the environment.

It has been confirmed that wheat-associated microbes are widely involved in plant Fe homeostasis, e.g., improving Fe uptake and alleviating Fe toxicity in wheat (12). Different microorganisms can not only increase yield production but also promote absorption and accumulation of certain essential elements in crop grains, a process termed microbial-assisted biofortification (13). In recent years, the use of microorganisms for enhancing wheat biofortification has attracted much attention (14). Microorganisms can significantly improve Fe accumulation in wheat in an efficient and eco-friendly way. Strains of *Bacillus* spp. form spores and are widely explored as plant growth-promoting bacteria (PGPB) in contemporary agriculture for different purposes (15, 16). They secrete siderophores, organic acids, and other compounds to promote the uptake of Fe in the rhizosphere of wheat (17, 18). Furthermore, they can improve the translocation or remobilization of Fe from the roots to the aerial parts and the accumulation in the grains (18, 19). *Bacillus* spp. has been widely recognized for its important role in helping plants obtain Fe to cope with Fe deficiency (20, 21). Field studies have demonstrated as high as a 70% increase of Fe content in wheat grains after inoculation with *B. pichinotyi* or *B. subtilis* (22, 23). In another study, the values of tillers per plant (TTP) and thousand-grain weight (TGW) increased more than 20%, and the levels of grain Fe increased more than 44% (24).

Due to a lower environmental impact and higher colonization ability in plants, endophytic bacteria may have better applicability than the widely used soil bacteria at present (25). We have isolated a series of endophytic bacteria from wheat roots (26). One of them, *B. altitudinis* WR10, has a strong ability to absorb Fe and improves the ability of wheat to tolerate Fe by upregulating the expression of wheat genes encoding ferritin (27). The strain has high phytase activity, produces siderophores, and forms biofilm (28). Therefore, this study was planned to inoculate wheat with this strain using different methods (such as soaking or spraying) to increase Fe content in wheat grains and achieve WR10-assisted Fe biofortification in wheat.

## Materials and Methods

### Bacterial Growth and Characterization

The strain *B. altitudinis* WR10 was previously isolated from the root of wheat (*Triticum aestivum* L. cv. Zhoumai 26) and stored in 20% glycerol at −80°C (27). The glycerol stock of *B. altitudinis* WR10 was streaked on Luria-Bertani (LB) agar. After overnight incubation at 30°C, a single colony was picked into 5 ml sterile LB broth in a glass tube. The tube was agitated at 30°C, 150 rpm for 24 h. For quantitative assay of hormone production, supernatants were collected by centrifugation at 8,000 g for 5 min. The concentrations of indoleacetic acid (IAA), cytokinin (CTK), and gibberellin (GA) in supernatants were assayed with commercial Plant IAA, CTK, or GA ELISA Kits using specific antibodies coated microplate (Enzyme-linked Biotechnology Co. Ltd., Shanghai), by reading absorbance at 450 nm (Abs.450 nm) and calibrating with corresponding standards. For qualitative assay of hydrolytic enzymes production, bacterial suspension was spotted on respective agars using starch, pectin, carboxymethylcellulose, or casein as the sole carbon source (29). A clear halo zone around spotted bacteria after staining indicates the production of corresponding enzymes. The production of hydrogen cyanide (HCN), ammonia, and siderophores was detected, as in a previous report (30). The intrinsic antibiotic spectra were tested on LB agar supplemented with different antibiotics, including ampicillin (100 μg/ml), chloramphenicol (5 μg/ml), erythromycin (5 μg/ml), kanamycin (50 μg/ml), and spectromycin (100 μg/ml). No growth after 24 h of incubation at 30°C was considered as sensitive. All these antibiotics are purchased at biotechnological grade (Sigma-Aldrich, USA). For pathogenicity analysis, the Virulence Factors Database (VFDB, www.mgc.ac.cn/cgi-bin/VFs/v5/main.cgi?fun=VFanalyzer) and the PathogenFinder (https://cge.cbs.dtu.dk/services/PathogenFinder) web-based tools were searched using the reference genome of *B. altitudinis* GR8 (31, 32). Strain GR8 has the highest identity in a marker gene *gyrB* of *B. altitudinis* WR10 (28).

### Microbial Inoculants Preparation, Field Application, and Wheat Planting

*Bacillus altitudinis* WR10 was cultivated in LB broth in 500 ml flasks, under 30°C, and agitating at 200 rpm. Cell pellets were collected from overnight culture fluids after centrifugation at 8,000 g for 5 min. Bacterial cells were washed two times with sterile water and then resuspended in water (10^7^ cfu/ml) for field application as microbial inoculants. For soil spraying, fresh inoculants were sprayed onto the surface of the soil using a sprinkling can (0.5 L per m^2^) 2 h before sowing. For seed soaking, wheat seeds (*Triticum aestivum* L. cv. Zhoumai 36) were immersed in fresh WR10 inoculants for 1 h under room temperature and agitating at 50 rpm. After incubation, seeds were dried by airing on a bench for 24 h. For both controls, equal volumes of water without bacteria were simultaneously used. The planting of the wheat was conducted in 2019–2020 at the Field Experimental Center of Zhoukou Normal University (N33°38′, E114°40′), China. Manipulation and application of *B. altitudinis* WR10 in the field were approved by the Institutional Biosafety Committee of Zhoukou Normal University. For each group, about 100 seeds (~5 g) were manually sowed in two lines, each with a length of 10 m. During growth, there was no extra fertilization or irrigation. Some chemical properties of the soil were assayed according to the respective national guidelines provided in the Supplementary Information (Supplementary Table 1).

### Wheat Sample Collection, Growth, and Yield Evaluation

At the grain filling stage (Feekes 11.1), 30 whole plants were collected randomly from different planting regions. Two growth parameters, including plant height and total chlorophyll content, were evaluated. Plant height above the ground was measured in centimeters. Then, plants were rinsed with tap water for 10 min to clean off any attachments. The clean plants were further cut into different sections, including the root, the stem, and the leaf (~5 cm in length). The content of chlorophyll in the leaves was quantified as described elsewhere (33). The total chlorophyll content was calculated according to the formula (20.21 × Abs.645+8.02 × Abs.663). The content was expressed as mg/g dry weight. At the maturity stage (Feekes 11.4), wheat spikes were harvested manually and stored in plastic bags. Among them, 30 spikes were hand thrashed and used for analyzing the number of kernels per spike (KPS) and TGW. The samples used for weighting were dried in a thermo-constant incubator at 60°C until completely dry.

### Hydroponic Coculture of Wheat Seedlings and *Bacillus altitudinis* WR10

Hydroponic coculture was carried out according to a previous report with minor modification (27). Briefly, 7-day-old well-grown seedlings of 60 of *Triticum aestivum* L. cv. Zhoumai 36 were planted in 6 plastic boxes each containing 1.2 L dH_2_O. These six boxes were allocated into two groups. For the coculture group, each box was supplemented with 1.2 ml concentrated suspension of *B. altitudinis* WR10 (10^9^ cfu/ml). For the control group, 1.2 ml autoclaved suspension of *B. altitudinis* WR10 was added to each box. After the addition of bacteria, seedlings were grown at a controlled temperature (25°C) in humid conditions (humidity 70%) under dark or light (12/12 h) for 48 h. Six seedlings from each box were collected at different time points, e.g., 0, 1, 2, 4, 6, 8, 10, 12, 24, and 48 h postinoculation (hpi).

### Quantification of Bacterial Abundance

The abundance of *Bacillus* spp. in different tissues, including the root, the stem, and the leaf collected at the grain filling stage was quantified by qPCR assays using genus-specific primers (B_groELF/B_groELR). The abundance of strain WR10 in wheat seedlings, either the root or the sprout, collected during hydroponic coculture was quantified by qPCR assays using strain-specific primers (qR10F/qR10R). For both quantifications, sections of different tissues were first ultra-sonicated for 5 min (2/2 s) in sterile distilled water at room temperature. Second, they were dried at 60°C until constant weight. Third, these wheat tissues from six plants were mixed and grounded in liquid nitrogen with a mortar. Fourth, total genomic DNA was extracted from 20 mg tissue powder using a HiPure Food Microbial DNA Kit according to the user guide (Magen, China). Finally, the abundance of *Bacillus* spp. was evaluated by quantifying the relative gene copy of *groEL*. The abundance of strain WR10 was evaluated by quantifying the relative gene copy of *GAPDH*. The qPCR reaction was conducted in a CFX96 Touch™ Real-Time PCR Detection System (BioRad, USA) using SYBR green qPCR Master Mix (Vazyme, China). The parameters for thermocycler and melting curves were the same as done previously (27). The sequences of the primers and the sizes of amplicons are supplied in the Supplementary Information (Supplementary Table 2).

### Assays of Nutrient Content in Different Wheat Tissues

Three macronutrients, namely total nitrogen (N), phosphorus (P), and potassium (K), and four micronutrients, namely Fe, zinc (Zn), manganese (Mn), and copper (Cu), were detected in different wheat tissues collected from the field. Tissues (0.1 g) used for macronutrients determination were digested by 5 ml H_2_SO_4_ and 2 ml H_2_O_2_ and diluted in 20 ml water. The dilute was neutralized with 10 M NaOH before being used for assays. The content of N was measured using the Kjeldahl method as previously described (34). The contents of P and K were measured with biochemical assay kits purchased from a company, Elabscience® (Wuhan, China). Precisely, the content of P was quantified by reading absorbance at 660 nm using the colorimetric assay kit (Cat. No. E-BC-K245-S). The content of K was quantified by turbidimetry assay at 450 nm using another kit (Cat. No. E-BC-K279-M). The concentrations of all micronutrients were assayed using a flame atomic absorption spectrophotometry (FAAS, Persee, China), as previously described (35). For wheat tissues and grains, a modified protocol was developed for sample processing based on a methodological report (36). Briefly, dry wheat tissues were milled into powder by a universal pulverizer or were grounded in liquid nitrogen with a mortar. Tissue powder was extracted by adding 0.5 M HNO_3_ (0.1 g tissue per 20 ml acid) in 50 ml plastic tubes shaking under 37°C at 200 rpm for 2 h. Supernatants were collected after centrifugation at 10,000 g for 5 min and were used for these assays in triplicates. Phytate content was assayed using a rapid colorimetric method described previously, which is based on the reaction between ferric ion and sulfosalicylic acid (37). Sodium phytate was used for the preparation of standard solutions (Sigma, Shanghai, China). Absorbance was read in microtiters at 500 nm for phytate assay. All spectrometric readings using microtiters were read by a SpectraMax i3x microplate reader (Molecular Devices, Sunnyvale, USA).

### Grain Iron Staining

Mature grains from different groups were carefully dissected longitudinally or transversely using a stainless steel surgical knife and stained for 1 h with Perls' Prussian blue staining solution (2% [w/v] potassium hexacyanoferrate [II] and 2% [v/v] hydrochloric acid) as described elsewhere (38). After being washed two times in distilled water, all stained grains were dried at room temperature for 1 h. The stained section of grains was observed using a stereo light microscope NSZ-405 (NOVEL, Ningbo, China) and images were acquired by Echoo Imager (OPLENIC, Hangzhou, China).

### Data Analysis

Original data were expressed as mean ± SD of at least three repeats. Statistical analysis was conducted in SPSS 19.0 (SPSS Inc., USA). Significant differences between groups were analyzed by one-way ANOVA using the LSD test. Values of *p* < 0.05 were considered statistically significant. Spearman's correlation coefficients were analyzed among bacterial abundance and different nutrient contents. The coefficient of determination (*R*^2^) was also calculated after linear regression using Microsoft Excel. To show data from different groups in different tissues in figures, they were normalized to corresponding values in the control group (NC). All related data can be found in the Supplementary Information.

## Results

### Characteristics and Potential Pathogenicity of *Bacillus altitudinis* WR10

Some characteristics of *B. altitudinis* WR10, including common PGP traits, were screened *in vitro* (Table 1). Briefly, ELISA assays using culture supernatants revealed the production of phytohormones, such as IAA and GA by strain WR10. The bacterium solubilized both inorganic (calcium phosphate) and organic (phytate) phosphorus. Production of ammonia and siderophores were also detected according to obvious phenotypes. Biofilm formation, exopolysaccharide secretion, and early colonization of wheat root were observed as well. For antagonistic traits, strain WR10 was sensitive to antibiotics, including ampicillin, chloramphenicol, erythromycin, kanamycin, and spectromycin under the tested concentrations. Strain WR10 was ACC deaminase positive and produced HCN. Regarding hydrolytic enzymes, strain WR10 produced amylase, cellulose, pectinase, and chitinase. Comprehensive analysis of pathogenicity factors by VFDB indicated the absence of most major virulence factors in *Bacillus* spp., albeit there were a few genes involved in capsule synthesis that may contribute to immune evasion. Detail results are provided in Supplementary Information (Supplementary Table 3). Further genome prediction by the PathogenFinder service confirmed strain WR10 as a non-human pathogen, as no matched pathogenic family was found.

**Table 1:** Production of enzymes and other characters of *B. altitudinis* WR10 related to plant growth-promoting (PGP) and antagonism.

**Character**	***B. altitudinis* WR10**
**PGP traits**	
IAA	31.5 pmol/ml
Cytokinin	–
Gibberelin	74.6 pmol/ml
Phosphorus solubilization	+
Siderophore production	+
Ammonia production	+
Biofilm formation	+
Exopolysaccharide	+
Early colonization	+
**Antagonistic traits**	
Antibiotic spectra	Amp^−^, Cm^−^, Erm^−^, Kan^−^, Spe^−^
HCN production	+
ACC deaminase	+
**Hydrolytic enzymes**	
Chitinase	+
Protease	–
Cellulase	+
Amylase	+
Pectinase	+

*+, positive; −, negative; ^−^, sensitive; Amp, ampicillin (100 μg/ml); Cm, chloramphenicol (5 μg/ml); Erm, erythromycin (5 μg/ml); Kan, kanamycin (50 μg/ml); Spe, spectromycin (100 μg/ml); HCN, hydrogen cyanide*.

### Effect of Microbial Inoculation on Yield and Nutrients of Wheat Grains

**Figure 1:**
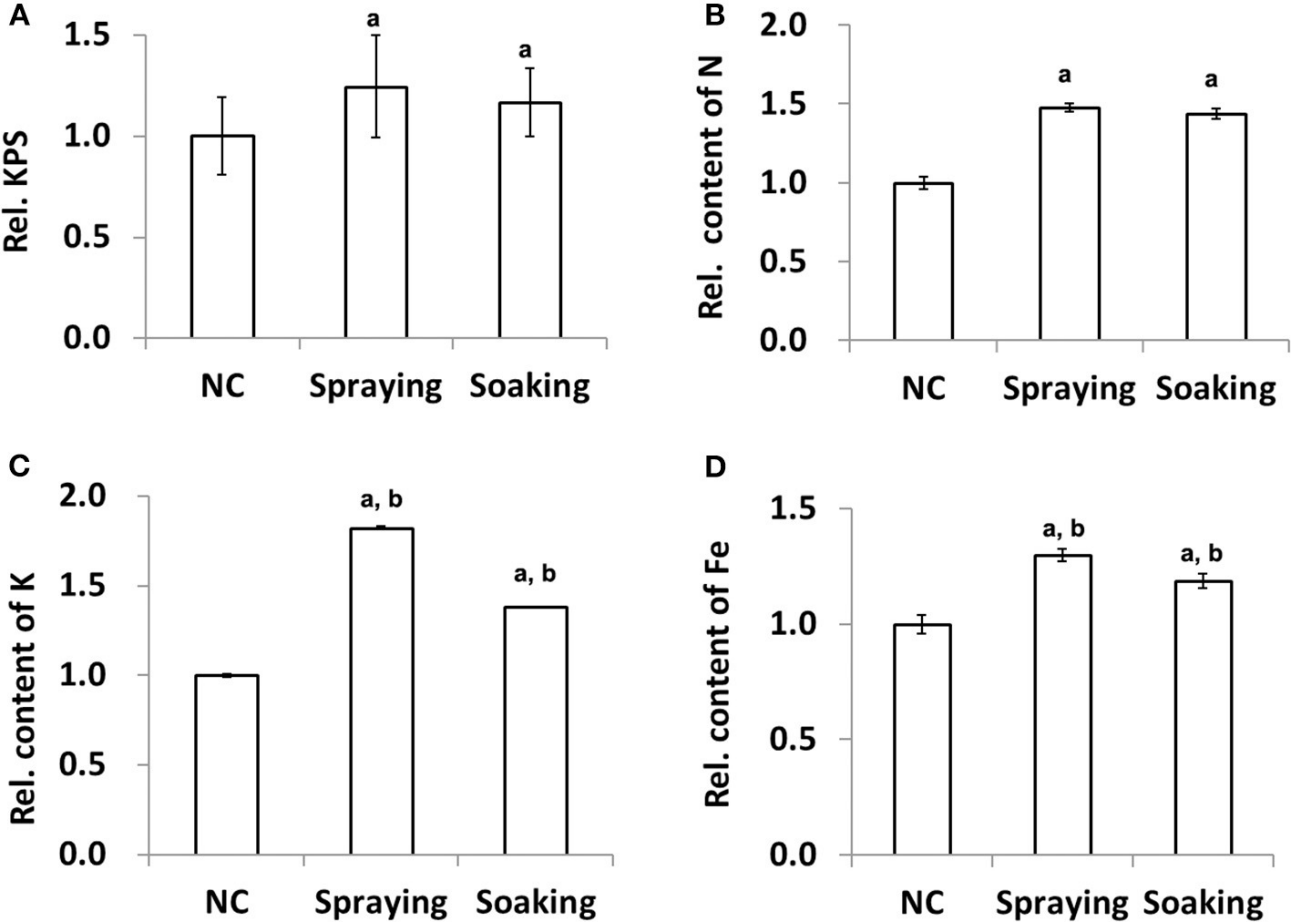
Effect of WR10 inoculation on wheat grain yield production and nutrient content. **(A)** The relative mean number of kernels per spike (KPS) of 30 wheat plants; **(B)** Relative content of total N in grains; **(C)** Relative content of K in grains; **(D)** Relative content of Fe in grains. Content of macronutrients was calculated as mg/g, and that of micronutrients was calculated as mg/kg dry weight (DW). Original data were analyzed by ANOVA pair-wise comparisons using LDS-test and *p* < 0.05 was considered significant. All data in the figure were normalized to their counterparts in the control group (NC) without inoculation of bacteria. In spraying, soils were sprayed with *B. altitudinis* WR10 before sowing of wheat; in soaking, wheat seeds were soaked in *B. altitudinis* WR10 suspension before sowing. a, statistically different from NC; b, significantly different between spraying and soaking groups.

After wheat harvest, the TGW and KPS were calculated for evaluating the impact of microbial inoculation on grain yield production. The data indicated that there was no change in TGW among the different groups (Supplementary Table 4). In contrast, the relative numbers of KPS were significantly larger in the two treated groups, e.g., increased by 24.67 and 16.44% in groups sprayed or soaked in WR10, respectively (Figure 1A). For macronutrients, quantification using whole wheat flour showed a significant increase of N and K contents, except P. For example, both total N and K contents were increased by more than 50% (Figures 1B,C). For micronutrients, there was no difference in the contents of Zn, Mn, and Cu after inoculation of WR10; however, Fe content was significantly increased by about 30 and 19% (exactly, 29.94 and 18.67%) in the spraying and soaking groups, respectively (Figure 1D). The absolute concentration of Fe in Zhoumai 36 was increased from 33.55 to 43.60 mg/kg in the spraying group. For all these changed indices except N, stronger effects were observed in the soil-spraying group than in the seed-soaking group. In addition, the phytate assay showed a slight decrease (~5%) in relative phytate content in the spraying group although there was no significant difference compared with the control (Supplementary Table 4).

### Effect of Microbial Inoculation on Wheat Vegetative Organs

**Figure 2:**
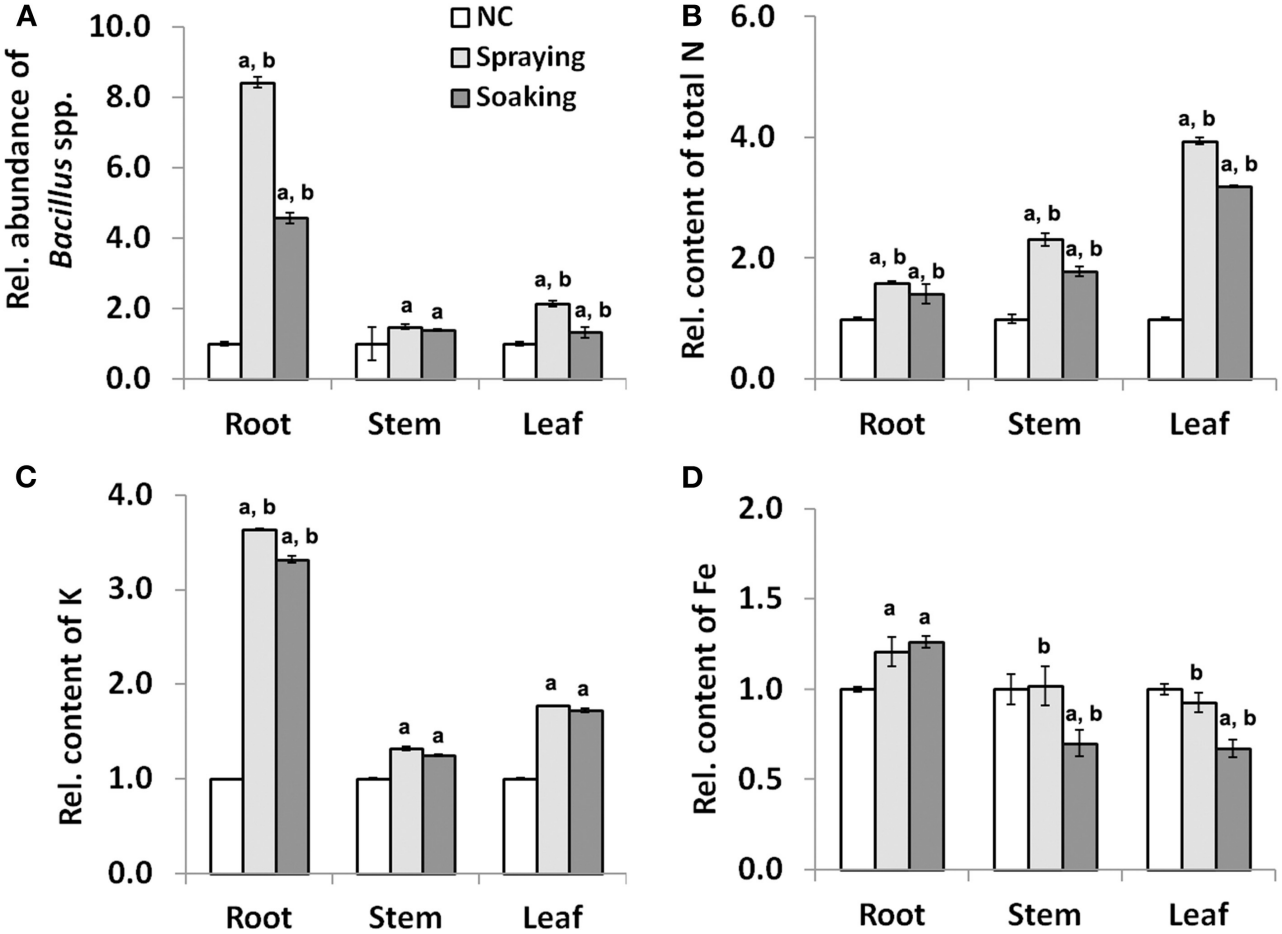
Effect of WR10 inoculation on the abundance of endophytic *Bacillus* spp. and nutrient content in different wheat tissues at the grain filling stage. **(A)** The relative abundance of endophytic *Bacillus* spp. in different wheat tissues; **(B)** Relative content of N in different wheat tissues; **(C)** Relative content of K in different wheat tissues; **(D)** Relative content of Fe in different wheat tissues. Content of macronutrients was calculated as mg/g, and that of micronutrients was calculated as mg/kg dry weight. Original data were analyzed by pair-wise comparisons using LDS test, and *p* < 0.05 was considered significant. All data in the figure were normalized to their respective counterparts in the control group (NC) without inoculation of bacteria. In spraying, soils were sprayed with *B. altitudinis* WR10 before sowing of wheat; in soaking, wheat seeds were soaked in *B. altitudinis* WR10 suspension before sowing; a, statistically different from NC; b, significantly different between spraying and soaking groups.

At the grain filling stage, two parameters were monitored to evaluate the impact of microbial inoculation on wheat growth (Supplementary Table 5). Compared with NC, there was no difference in plant height among the three groups; however, there was a significant increase in total chlorophyll content in leaves collected from the two inoculated groups. Precisely, the content of total chlorophyll was increased by 42.07 and 22.85% in groups sprayed or soaked WR10, respectively. The qPCR quantification of *Bacillus* spp. showed a positive influence of microbial inoculation as the relative abundance was always higher after the inoculation of *B. altitudinis* WR10 (Figure 2A). In particular, the abundance of *Bacillus* spp. increased more than 7- or 3-fold in the root after being sprayed or soaked with WR10, respectively. In both the root and the leaf, the relative abundance of *Bacillus* spp. was higher in the spraying group than in the soaking group, suggesting a stronger influence of the former application method. Regarding nutrients, on the one hand, the inoculation of WR10 constantly increased the contents of N and K in all tested tissues, including the root, the stem, and the leaf (Figures 2B,C). On the other hand, the inoculation with WR10 had different impacts on Fe content in different tissues. In general, inoculation with WR10 significantly increased the relative content of Fe in the root but decreased the relative content in the stem and the leaf (Figure 2D, Supplementary Table 6).

### Iron Staining and the Relationship Between *Bacillus* spp. Abundance and Nutrient Content

**Figure 3:**
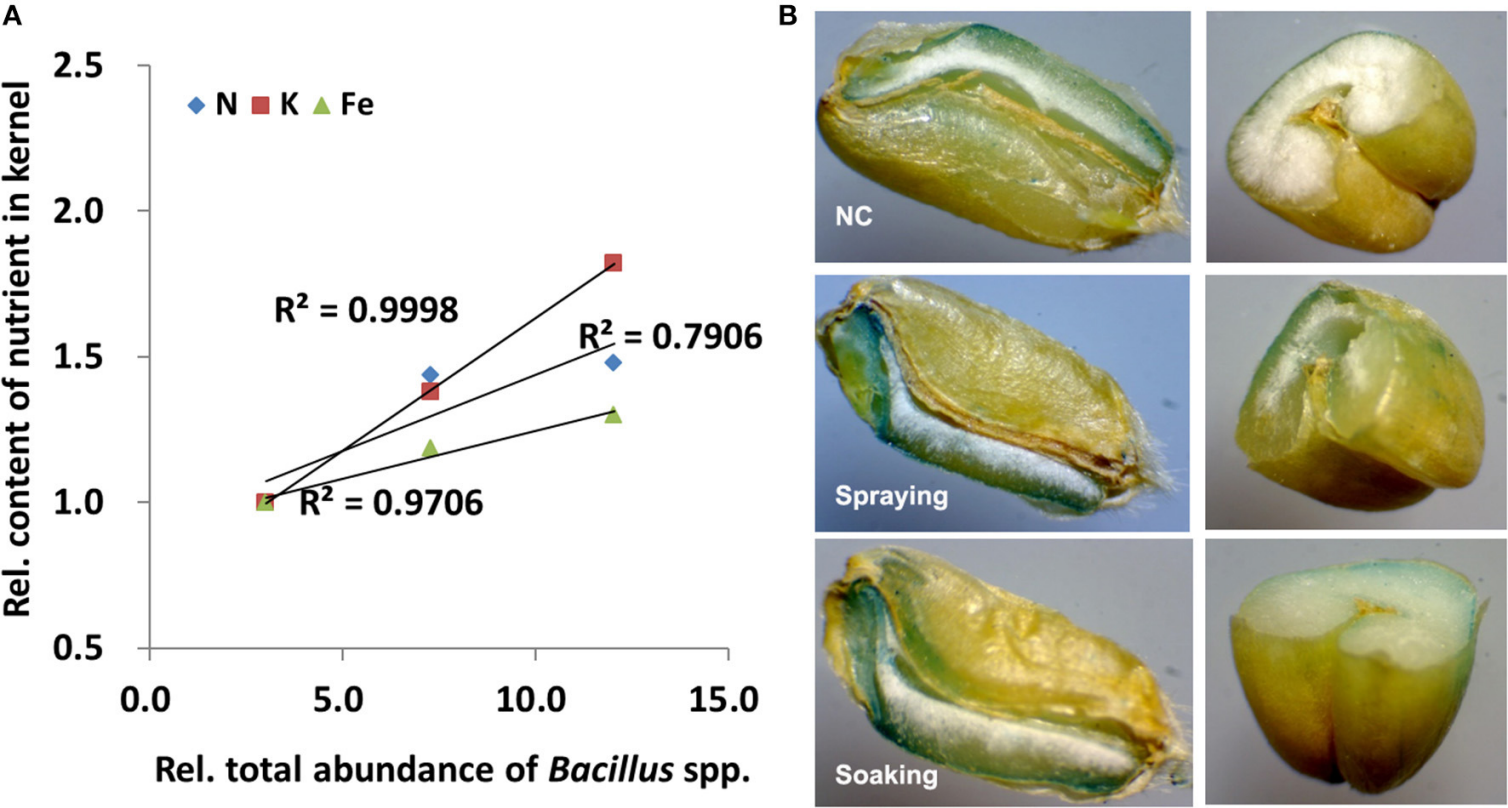
Correlation analysis and iron staining. **(A)** Linear regression and coefficients of determination (*R*^2^) between the total abundances of *Bacillus* spp. and content of three nutrients; **(B)** Perls' Prussian blue staining of grains. All data were normalized to their respective counterparts in the control group (NC) without inoculation of bacteria. In spraying, soils were sprayed with *B. altitudinis* WR10 before sowing of wheat; in soaking, wheat seeds were soaked in *B. altitudinis* WR10 suspension before sowing.

Spearman's correlation analysis showed that a positive relationship exists between the changed nutrient contents and the abundance of endophytic *Bacillus* spp. (Supplementary Table 7). In particular, Spearman's correlation coefficient was 0.937 or 0.933 for K or Fe and bacterial abundance (*p* < 0.01). As shown in Figure 3A, the nutrient content in grains has a high linear correlation to the total abundance of *Bacillus* spp. in all vegetative organs. For example, the *R*^2^ between either K or Fe content and the total abundance of *Bacillus* spp. is higher than 0.9. To further investigate the distribution of Fe in grains, Fe staining was performed. Whether dissected longitudinally (left panel) or transversely (right panel), Perls' Prussian blue staining of grains showed the distribution of iron (Figure 3B). In contrast to NC, much intense blue staining could be observed in aleurone, embryo, and endosperm from grains harvested in the spraying and the soaking groups.

### The Colonization, Internalization, Translocation, and Replication of *B. altitudinis* WR10 in Wheat

**Figure 4:**
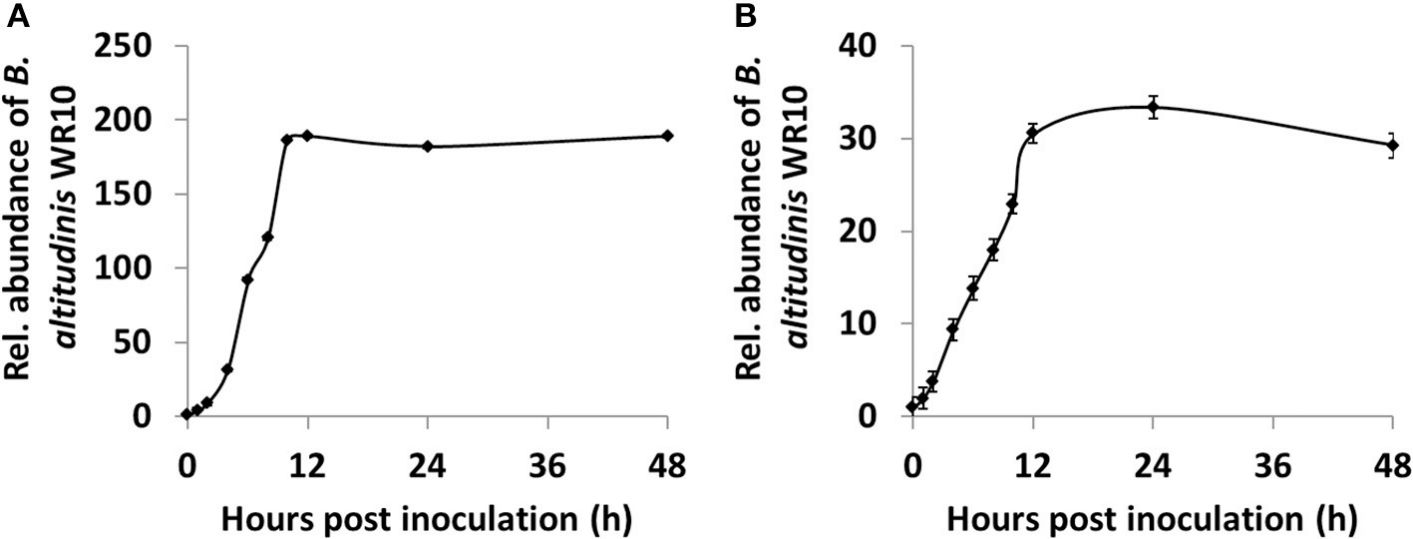
Quantification of *B. altitudinis* WR10 in different wheat tissues during hydroponic coculture by qPCR assay. **(A)** The relative abundance of endophytic *B. altitudinis* WR10 in the root; **(B)** relative abundance of endophytic *B. altitudinis* WR10 in the sprout. At 0 phi, the relative abundances in different tissues were considered as 1. Fold changes were calculated by the 2^ΔCq^ method, which uses the copy numbers of the *GAPDH* gene representing the abundance of *B. altitudinis* WR10. Data were mean of three repeats from the genomic DNA mixture of six seedlings.

In the control group, *B. altitudinis* WR10 could not be detected by qPCR from all wheat samples, indicating its absence in Zhoumai 36; however, it could be constantly detected from seedlings of Zhoumai 36 as endophyte, after its addition for hydroponic coculture (Figure 4). To be precise, within 1 h (1 hpi), WR10 could even be detected in the root, suggesting its fast colonization and internalization. After that, the relative abundance of WR10 was improved sharply by nearly 200-fold within 10 h (10 hpi), indicating quick internalization and/or replication; however, the abundance of WR10 reached a plateau after 10 hpi and remained constant within the tested periods (Figure 4A). Similarly, WR10 could be detected in the sprout at 1 hpi, suggesting its quick translocation from the root. Further, the relative abundance of WR10 also increased steadily by more than 30-fold within 24 hpi, indicating continuous translocation and/or replication (Figure 4B). In addition, the abundance of WR10 was always much lower in the sprout than in the root.

## Discussion

Some wheat-associated microbes, mainly the rhizospheric microbes, produce siderophores and other metabolites that increase Fe solubility in the soil and can alleviate Fe-deficiency stress in the plant (12). Therefore, these microbes have been proposed as biofertilizers for enhancing Fe acquisition of crops due to their important role in favoring plant-iron uptake and accumulation under limiting conditions (39). Indeed, through inoculation of versatile microorganisms, enhanced uptake of Fe in wheat had been achieved by many previous studies (14); however, the majority of microbes used have been soil microorganisms and Fe concentration was only tested in the root. Hence, the stability and the effectiveness in practice are highly variable. In this study, we used an endophytic bacterium, *B. altitudinis* WR10, because it has a strong ability to absorb Fe and improves the ability of wheat to tolerate Fe (27). In addition, the strain produces siderophores and secretes phytase (28). These properties made the strain a good candidate for assisting wheat Fe biofortification for three reasons at least. First, WR10 can improve Fe bioaccessibility in soils and improve Fe accumulation in tissues. Second, it can decrease Fe toxicity within wheat tissues. Third, WR10 may degrade phytate, thereby improving Fe bioavailability in grains, which is important from the perspective of human nutrition.

To apply the strain, we first evaluated its pathogenicity and several PGP traits (Table 1). Unsurprisingly, strain WR10 was predicted as a non-human pathogen. Considering the strain was isolated from the root of healthy wheat as an endophyte, it should also be non-pathogenic to plants and can be used in agricultural systems. The characteristics listed in Table 1 suggest that the strain possesses many growth-promoting and antagonistic properties. All these data support its potential application without unseen biosafety concerns (29, 30). As a pilot field experiment, this study tested the potential of *B. altitudinis* WR10 in natural field conditions, without any fertilization or irrigation.

The influence of microbial inoculation on yield and nutrients of grains was first evaluated, as improving yield is always the primary target of wheat planting. A few field studies have demonstrated the positive effect of microbial inoculants on wheat yield (22, 40, 41). It has been reported that stress-tolerant *Viridibacillus arenosi* strain IHB B7171 enhances grain yield by 13.9% in wheat (42). Yadav et al. reported a more than 20% increase in the values of TTP and TGW by inoculation with *Bacillus subtilis* CP4 and Arbuscular mycorrhizal fungi (AMF) (24, 43). This study demonstrated a significant increase in the number of KPS (16.44 or 24.67%) after bacterial inoculation, without influence on TGW (Figure 1A, Supplementary Table 4). Improvement in the number of KPS has also been reported in wheat after inoculation with AMF (44); however, the real effect of microbial inoculants on total grain yield production still needs comprehensive investigation, as this is often restrained by multiple factors. For example, grain yield is more significantly affected by nitrogen fertilization than AMF inoculation (44). Furthermore, inoculation with an endophytic nitrogen-fixing bacterium, *Paraburkholderia tropica*, had shown little effect on wheat grain yield, either with or without fertilization (45).

Except for increasing yield, the use of microbes for improving nutrient acquisition has also been evaluated across a variety of crops under varying conditions (46). It has been shown that AMF inoculation has a positive effect on Cu, Fe, and Zn content in all tissue types of wheat (40). As high as a 70% increase in Fe content in wheat grains after inoculation with *B. pichinotyi* or *B. subtilis* has been reported (22, 23). In another study, *B. subtilis* CP4 and AMF in combination increased Fe content in wheat grains by more than 44% (24). To fully discover the effect of WR10 application on grain nutrients, we evaluated three macronutrients and four micronutrients in grains. Data demonstrated that the one-time application of *B. altitudinis* WR10 significantly improves the content of N, K, and Fe (Figure 1). Especially, among all tested micronutrients, Fe content in grains was increased by about 30 and 19% in the spraying and soaking groups, respectively; however, there was no change in P, Zn, Mn, and Cu (Supplementary Table 4). In addition, we assayed phytate content in grains. Phytate is widely recognized as anti-nutritional because of the strong binding potential with minerals, including Fe and Zn (47). Strain WR10 produces phytases that effectively degrade phytate (28). Therefore, the content of phytate can be decreased after inoculation with WR10; however, this data suggest that this is not true in grains (Supplementary Table 4), and, as reported in AMF, the positive effect of WR10 on plant Fe accumulation may also be modulated by wheat genotypes, soil pH, texture, and nutrient concentration, as well as agronomic practices, such as N and P fertilization (46, 48).

Due to the importance of plant growth on yield production and nutrient accumulation in grains, we monitored plant height and total chlorophyll content in leaves at the grain filling stage, a vital phase for wheat kernel development. Plant height was measured as the first index due to its crucial role in plant architecture and yield potential (49); however, plant height showed no difference among the different groups (Figure 1A). The results agreed with a study that tested 13 single-inoculated bacteria, in which the plant height of wheat seedlings was measured after growing in pots for 80 days (50). Second, it was revealed that plant chlorophyll content is positively correlated with nitrogen content, which is important for crop quality and yield (51, 52). Hence, the total content of chlorophyll is a good indicator of the nutritional status of the plant and has significance for modern precision agriculture in practice. In this study, inoculation with WR10 significantly increased the total content of chlorophyll in leaves, indicating its positive effect on wheat nutrition and potentially on yield production (Figure 1B). In line with our observation, a large number of studies have reported an increase in chlorophyll content or a decrease in its loss by different PGPB in wheat grown under various conditions (53–56).

Although there is no doubt that microbes play an important role in plant nutrition, quantitative estimations of microbial-plant interactions are still scarce, especially under field conditions (57). By quantification of the abundance of endophytic *Bacillus* spp., a major group of bacteria explored in contemporary agriculture, this study showed a complex effect of microbial inoculation on a certain genus within different tissues of wheat (Figure 2A). At the same time, quantification of Fe content in different tissues provided insights into how Fe hemostasis in the plant is regulated by bacteria. For example, at the grain filling stage, Fe content was improved in the root but decreased in the stem and the leaf (Figure 2B). The results indicate microbial inoculants, like *B. altitudinis* WR10, may enhance Fe uptake of roots from soils and strengthen Fe translocation in stems and remobilization in leaves. Therefore, much Fe can be acquired in the root, with less Fe in the stem and the leaf after inoculation of bacteria. Taken together, inoculation with *B. altitudinis* WR10 improved the abundance of *Bacillus* spp., which in turn improved Fe accumulation in grains, mainly by increasing Fe acquisition in roots from soils.

In addition, the formulation and application method showed an obvious impact on the effect of microbial inoculants (58). As a pilot study, we evaluated the two most widely applied methods and did not consider formulation in this study. Nearly, all indicators showed that liquid soil spraying has a stronger influence than seed soaking (Figures 1, 2). For example, the abundance of *Bacillus* spp. was higher in all tissues in the spraying group than in the soaking group. This might be because more *B. altitudinis* WR10 were introduced into soils by soil spraying than by seed soaking or WR10 replicated/colonized much more easily in the former application method. Although it was reported that all inoculation methods, including in-furrow inoculation, soil spraying, foliar spraying, and seed soaking of *Azospirillum brasilense* increased the abundance of diazotrophic bacteria in wheat tissues, soil inoculations favored root and rhizosphere colonization (59). Root and rhizosphere colonization is important for the function of inoculants, as Fe can only be absorbed by the roots from the rhizospheric soil (12). In tobacco plants, soil inoculation led to pronounced bacterial-induced effect than seed inoculation in a mine soil contaminated with heavy metals (60). In Italian ryegrass, it was also showed that soil spraying performed better than seed soaking using different microbial inoculants, which further showed that the beneficial effect was correlated with the colonization efficiency of the inoculated strains (61). Therefore, it seems soil spraying is a more effective method for microbial inoculation than seed soaking, especially considering the feasibility and stability at a commercial scale (62). Furthermore, this study demonstrated a high correlation between the total abundance of *Bacillus* spp. in all vegetative organs and some nutrient content in grains (Figure 3A). A positive correlation exists between the total abundance of *Bacillus* spp. and the contents of N, K, and Fe in grains (Figure 3A, Supplementary Table 7). The result is reasonable as nutrient accumulation in grains is determined by both uptakes in roots, translocation in stems, and remobilization/distribution in leave; however, different regulations by the same bacterium can be seen in different nutrients (Figure 2). For example, in all tissues, the contents of N and K were higher along with a higher abundance of *Bacillus* spp. In contrast, Fe content was higher in the root but lower in leaves when having more *Bacillus* spp; however, the complex and different regulation of nutrient content in different wheat tissues by microbial inoculants are frequently reported in previous studies (13, 22–24, 43).

To quantify the relative abundance of the inoculant WR10, a hydroponic coculture model was used. A sharp increase in the relative abundance of WR10 was detected by qPCR assays in both the root and the sprout after bacterial inoculation, contrasting with uninoculated controls (Figure 4). The results indicated quick and efficient colonization, internalization, translocation, and/or replication of *B. altitudinis* WR10 in Zhoumai 36. Indeed, the relative abundance of WR10 is always much higher in the root than in the sprout at the same time point and effective colonization of AMF, as well as other PGPB, has also been reported in the root of wheat (24, 44); however, this experiment provided more information regarding the quantitative or dynamic distribution of microbial inoculants in different tissues of wheat. It was also shown that exogenous WR10 reached a plateau in both the root and the sprout after a certain number of hours (e.g., 10 or 12 hpi).

In summary, strain *B. altitudinis* WR10 is a non-pathogen with versatile PGP and antagonistic traits. The strain can efficiently colonize and translocate within wheat. Its inoculation significantly enhances Fe biofortification in wheat grains (*Triticum aestivum* L. cv. Zhoumai 36) in the field, prospecting a promising potential for further investigation of WR10-assisted Fe biofortification. Also, Fe content in grains was positively correlated with the total abundance of endogenous *Bacillus* spp. in wheat. In addition, soil spraying is much more effective than seed soaking in increasing grain Fe content. To pave the way for microbial-assisted biofortification, the influence of application routines, soil chemicals, fertilization regimes, as well as the genotypes of wheat also need to be evaluated in the future.

## Data Availability Statement

The raw data supporting the conclusions of this article will be made available by the authors, without undue reservation.

## Author Contributions

ZS and CL designed the experiment, wrote the manuscript, discussed the results, and finalized the manuscript. ZS, ZY, and HL carried out the experiments. ZS and KM analyzed the data. All authors contributed to the article and approved the submitted version.

## Conflict of Interest

The authors declare that the research was conducted in the absence of any commercial or financial relationships that could be construed as a potential conflict of interest.

## Publisher's Note

All claims expressed in this article are solely those of the authors and do not necessarily represent those of their affiliated organizations, or those of the publisher, the editors and the reviewers. Any product that may be evaluated in this article, or claim that may be made by its manufacturer, is not guaranteed or endorsed by the publisher.
