# Supplementary material for: Microbial-Assisted Wheat Iron Biofortification Using Endophytic Bacillus altitudinis WR10
Source: Front Nutr. 2021 Aug 3;8:704030. doi: 10.3389/fnut.2021.704030 (PMC8368724; doi:10.3389/fnut.2021.704030)
Supplement: Supplementary file 1 [file Table_1.DOCX]

**Microbial-assisted wheat iron biofortification using endophytic *Bacillus altitudinis* WR10**

Zhongke Sun^1, 2, *^, Zonghao Yue^1^, Hongzhan Liu^1^, Keshi Ma^1^, Chengwei Li^2, *^

1. College of Life Science and Agronomy, Zhoukou Normal University
2. College of Biological Engineering, Henan University of Technology

Corresponding to

Prof. Chengwei Li, Email: [lcw@haut.](mailto:lcw@haut.)edu.cn or

Dr. Zhongke Sun, Email: [sunzh@daad-alumni.de](mailto:sunzh@daad-alumni.de),

ORCID: https://orcid.org/0000-0002-9784-9769

Post address: Room A407, Life Sciences Building, Zhoukou Normal University, Wenchang Road, Zhoukou, 466001, China

Tel/Fax: 86-0394-8178259

# Supplementary Information

Table S1 Basic properties and nutritional indices of the soil

| Test item | Result | Reference method |
| --- | --- | --- |
| Total nitrogen | 1.10g/kg | LY/T 1228-2015 3.1 |
| Total phosphorus | 0.42g/kg | LY/T 1232-2015 3.2 |
| Total potassium | 1.53% | NY/T 87-1988 |
| Organic carbon | 0.54% | HJ 615-2011 |
| Organic matter | 9.38g/kg | NY/T 1121.6-2006 |
| Available phosphorous | 9.78mg/kg | LY/T 1232-2015 |
| Available potassium | 104mg/kg | LY/T 1234-2015 |
| Ammonia nitrogen | 2.76g/kg | HJ 634-2012 |
| pH | 8.20 | HJ 962-2018 |

Note: data were mean of five samples collected randomly from the planting area.

Table S2 Sequences of primers and sizes of amplicons

| Target gene | Primer sequence | Amplicon size | Annotation |
| --- | --- | --- | --- |
| *groEL* | B_groELF: CGTATCCGGTGGTGGTACAG  B_groELR: GCGATTTGACGGATTGGCTC | 129bp | Genus specific primers for qPCR |
| *GAPDH* | qR10F: TTCGTTGTCATACCACGCCA  qR10R: ACCGCTCGTTTCGACTGATT | 115bp | Strain specific primers for qPCR |

Table S3 Analysis of the pathogenicity factors by VFDB using a reference genome

| VFclass | Virulence factors | Related genes | *B. altitudinis* GR-8 (CP009108) |
| --- | --- | --- | --- |
| Adherence | Fibronectin-binding protein(Listeria) | fbpA | ID12_12805 |
| Enzyme | Immune inhibitor A metalloproteinase | inhA | ID12_04210 |
| Immune evasion | Hyaluronic acid (HA) capsule | hasB, basC | ID12_02680, ID12_14540 |
|  | Polyglutamic acid capsule | capA-D | ID12_02585, ID12_02595, ID12_02590, ID12_11260 |
|  | Polysaccharide capsule | Undetermined | ID12_01700; ID12_01710; ID12_01750; ID12_02470; ID12_03640 |
| Toxin | Hemolysin III | hlyIII | ID12_15125 |
| Invasion | Lipoprotein promoting entry protein (Listeria) | lpeA | ID12_01410 |
| Lipid and fatty acid metabolism | Pantothenate synthesis (Mycobacterium) | panD | ID12_15425 |
| Peptidoglycan modification | OatA (Listeria) | oatA | ID12_04260 |
| Surface protein anchoring | Lipoprotein diacylglyceryl transferase (Listeria) | lgt | ID12_02030 |
|  | Lipoprotein-specific signal peptidase II (Listeria) | lspA | ID12_12705 |
|  | Sortase B (Listeria) | srtB | ID12_03915 |

Note：Only potential virulence factors were presented based on the prediction by the Virulence Factors Database (http://www.mgc.ac.cn/VFs/).

Table S4 Effect of *B. altitudinis* WR10 inoculation on wheat grain yield production and nutrient content

| Group | NC | Spraying | Soaking |
| --- | --- | --- | --- |
| KPS | 43.5±8.29 | 54.1±11.02^a^ | 50.65±7.35^a^ |
| TGW (g) | 49.48±1.01 | 50.51±1.38 | 50.08±0.67 |
| N (mg/g) | 37.58±0.72 | 60.90±1.02^a^ | 58.85±0.39^a^ |
| P (mg/g) | 4.81±0.06 | 5.06±0.04 | 4.83±0.01 |
| K (mg/g) | 3.79±0.03 | 6.54±0.02^a, b^ | 5.06±0.01^a, b^ |
| Fe (mg/kg) | 33.55±1.34 | 43.60±0.89^a, b^ | 39.81±1.11^a, b^ |
| Zn (mg/kg) | 45.67±2.21 | 45.96±0.50 | 48.57±0.91 |
| Mn (mg/kg) | 3.38±0.06 | 3.20±0.07 | 3.28±0.06 |
| Cu (mg/kg) | 2.56±0.21 | 2.44±0.05 | 2.44±0.05 |
| Phytate (mg/g) | 26.06±1.17 | 24.78±1.11 | 25.83±1.16 |

Note: Significant differences between groups were analyzed by one-way analysis of variance (ANOVA) using LSD-test. The *p* < 0.05 was considered significant. NC, negative control that without inoculation of bacteria. Spraying, soils were sprayed with *B. altitudinis* WR10 before sowing of wheat; Soaking, wheat seeds were soaked in *B. altitudinis* WR10 suspension before sowing. KPS, kernels per spike; TGW, thousand grains weight; N, nitrogen; P, phosphorus; K, potassium; Fe, iron; Zn, Zink; Mn, Manganese; Cu, copper; a, statically different from NC; b, significantly different between Spraying and Soaking groups

Table S5 Effect of *B. altitudinis* WR10 inoculation on wheat growth at the grain filling stage

| Group | NC | Spraying | Soaking |
| --- | --- | --- | --- |
| PH | 74.6±5.3 | 75.2±9.4 | 75.5±5.9 |
| TChl (mg/g) | 5.56±0.04 | 7.89±0.23^a, b^ | 6.83±0.46^a, b^ |

Note: Significant differences between groups were analyzed by one-way analysis of variance (ANOVA) using LSD-test. The *p* < 0.05 was considered significant. NC, negative control that without inoculation of bacteria. Spraying, soils were sprayed with *B. altitudinis* WR10 before sowing of wheat; Soaking, wheat seeds were soaked in *B. altitudinis* WR10 suspension before sowing. PH, the plant height above ground; TChl, total chlorophyll content in dry leaves; a, statically different from NC; b, significantly different between Spraying and Soaking groups

Table S6 Effect of *B. altitudinis* WR10 inoculation on *Bacillus* spp. abundance and nutrient content at the grain filling stage

| Item | Tissue | NC | Spraying | Soaking |
| --- | --- | --- | --- | --- |
|  | Root | 1.00±0.04 | 8.44±0.86^a, b^ | 4.58±0.46^a, b^ |
| Rel. Abu | Stem | 1.04±0.32 | 1.48±0.07 ^a^ | 1.38±0.02 ^a^ |
|  | Leaf | 1.00±0.05 | 2.14±0.13^a, b^ | 1.33±0.14^a, b^ |
|  | Root | 25.78±0.43 | 46.96±0.60^a, b^ | 40.43±4.53^a, b^ |
| N (mg/g) | Stem | 3.64±1.03 | 22.88±1.20^a, b^ | 15.14±1.16^a, b^ |
|  | Leaf | 6.96±0.3 | 56.03±0.31^a, b^ | 43.62±0.25^a, b^ |
|  | Root | 3.49±0.01 | 4.96±0.02^a, b^ | 4.55±0.04^a, b^ |
| K (mg/g) | Stem | 2.70±0.03 | 3.50±0.05 | 3.32±0.02^a^ |
|  | Leaf | 3.47±0.03 | 5.98±0.01^a^ | 5.80±0.06^a^ |
|  | Root | 209.69±9.24 | 252.04±6.64^a^ | 263.30±14.4^a^ |
| Fe (mg/kg) | Stem | 55.59±2.54 | 56.43±1.73^b^ | 39.90±1.51^a, b^ |
|  | Leaf | 106.81±3.57 | 99.35±6.80^b^ | 72.97±2.79^a, b^ |

Note: Significant differences between groups were analyzed by one-way analysis of variance (ANOVA) using LSD-test. The *p* < 0.05 was considered significant. NC, negative control that without inoculation of bacteria. Spraying, soils were sprayed with *B. altitudinis* WR10 before sowing of wheat; Soaking, wheat seeds were soaked in *B. altitudinis* WR10 suspension before sowing. Rel. Abu, relative abundance of *Bacillus* spp.; N, nitrogen; K, potassium; Fe, iron; a, statically different from NC; b, significantly different between Spraying and Soaking groups

Table S7 Spearman correlation analysis between the content of nutrient and the abundance of *Bacillus* spp..

| Variables | Correlation coefficient | *p* value |
| --- | --- | --- |
| N content vs. bacterial abundance | ρ =0.767* | 0.016 |
| K content vs. bacterial abundance | ρ =0.937** | 0.000 |
| Fe content vs. bacterial abundance | ρ =0.933** | 0.000 |

Note: Bacterial abundance is the relative total abundance of *Bacillus* spp. in the root, stem and leaf at the grain filling stage. N content, relative nitrogen content in grains; K content, relative potassium content in grains; Fe, relative iron content in grains; *, *p* < 0.05; **, *p* < 0.01
